# Multimodal Treatment With ECT for Identity Integration in a Patient With Dissociative Identity Disorder, Complex Post-traumatic Stress Disorder, and Major Depressive Disorder: A Rare Case Report

**DOI:** 10.3389/fpsyt.2018.00275

**Published:** 2018-06-21

**Authors:** Kyle D. Webster, Susan Michalowski, Thomas E. Hranilovich

**Affiliations:** ^1^College of Osteopathic Medicine, Michigan State University, East Lansing, MI, United States; ^2^Psychiatric Associates, Okemos, MI, United States

**Keywords:** dissociative identity disorder, personality integration, post-traumatic stress disorder, major depressive disorder, suicidal ideation, electroconvulsive therapy

## Abstract

The legitimacy and etiology of Dissociative Identity Disorder (DID) remains a controversial topic within Psychiatry. The two schools of thought are the Post-Traumatic Model (PTM) and the Socio-Cognitive Model (SCM). This case highlights the validity of PTM in an individual who suffered severe and prolonged physical, psychological, and sexual abuse from 2 years old through adulthood. The reported abuse was corroborated and proven on two separate occasions via medical professionals/rape kit and the police. This resulted in the incarceration of one of her abusers. The only way for the patient to cope with the trauma she suffered was to dissociate, which resulted in the development of four full identity alters. In addition to being diagnosed with DID, the patient has been diagnosed with Major Depressive Disorder (MDD), Post-Traumatic Stress Disorder (PTSD), and chronic suicidality. Unable to manage the suicidal ideations and MDD after nearly 10 years of therapy and psychiatric medications, the patient was referred for Electroconvulsive Therapy (ECT). Upon receiving ECT weekly for 2 years, the patient reported having “lost the others.” As ECT progressed she went from having four alters to no alters and at the time of this report only being able to vaguely hear alter #4. With the integration of these alters she had access to the memories and pain that the alters had protected her from. Prior to losing the alters, her long-term memory was impaired by dissociative processes. Her long-term memory was also impaired because when one of the alters was in control of consciousness only that alter remembered what had happened during that time, unless that alter shared what had happened with one or more of the others. It is unclear if frequent ECT was the catalyst that lead to the integration of her alters however, integration finally began following prolonged ECT. This case highlights the importance of the PTM as an etiological description for DID and the importance of mental health providers further studying and researching the effects of ECT on patients with chronic MDD, PTSD, and suicidal intent, especially if these are comorbid with DID.

## Introduction/case presentation

A currently 39-year-old European-American female sought mental health treatment, starting 10 years ago, per her husband's request after she tried to kill herself. She was originally diagnosed with Major Depressive Disorder (MDD) severe with Mood-Congruent Psychotic Features, and Post-Traumatic Stress Disorder (PTSD) with Dissociative Symptoms. The original diagnosis of PTSD was established from familial physical abuse that the patient suffered. The original diagnoses were given after the patient had been hospitalized multiple times in her 20's but it wasn't until she became chronically suicidal that she sought regular psychiatric assistance.

After years of psychiatric and psychological treatment, with minimal efficacy, the patient admitted to having “others” who lived in her head and sometimes took over her consciousness. The psychologist treating her diagnosed the patient with Dissociative Identity Disorder (DID) because the patient met the criteria in accordance with the DSM V. The psychologist obtained more extensive training on treating DID patients and offered the patient the option to consult another therapist and she declined due to their trusting therapeutic relationship.

It was discovered that the patient had suffered continuous psychological, physical, and sexual abuse at the hands of various family members, friends of the family, and strangers beginning when she was a toddler and through early adulthood. Two of the instances of sexual abuse were documented in a local hospital one of them lead to the incarceration of the patient's uncle. Furthermore, the patient admitted that her mother knew of some of the abuse with her family while she was a minor but her mother decided not to report the abuse and keep it within the family. As a result of being told her whole life to keep secrets by her family, the patient took extra care in hiding the existence of her alters. This resulted in the difficulty in identifying the alters and was only possible once a trusting therapeutic relationship was established between the patient and her psychologist. The therapy administered to the patient was considered eclectic psychotherapy, that included, but was not limited to: psychodynamic psychotherapy, hypnotherapy, existential-humanistic, problem-centered, and cognitive behavioral therapy. In over 450 sessions the patient's trauma and alters were gradually documented and elucidated.

The host personality was hard to identify and was originally thought to be an alter. The host personality did not go by the patient's birth name, but rather by a name given by the other alters. In daily life, the host personality and alters all go by the patient's birth name even though they each have their own. The host personality is the only one that has aged with the physical body and is the proper chronological age.

The four full personality alters (#1–#4) developed from repeated trauma, each displaying separate and distinct characteristics when dominating her consciousness and having separate memories, emotions, and skills. Number 1 came into existence to protect the patient from trauma when the patient was no more than 3 years old and the abuse and assault by her uncle escalated significantly. The shyest of the alters, #1, held the memories and emotions stemming from trauma during the time the patient was between 3 and 8 years old, and stopped aging when the patient was eight when #2 came into existence and remained that age. Number 2 came into existence when the patient revealed the abuse and assault by her uncle. This alter protected the patient from the trauma of talking about and testifying against her uncle, being forced to visit him in prison, and write him letters of apology by her family for having “lied” about the abuse even though the sexual assault was documented by ER personal. Number 2 aged with the patient until she was around 10 years of age and stopped aging when #3 came into existence and has remained that age. Following her uncle's incarceration, the patient had court ordered therapy however, her mother didn't see value in therapy and removed the patient after a few sessions. Number 3 came into existence when the patient's uncle was paroled and protected the patient until she was 12 years old. Number 3 took control of consciousness when the patient had sex and whenever this alter wanted to have a “good time,” e.g., get drunk and/or have sex. The last alter, Number 4, came into existence when the patient was 12 years old and would exchange sex for drugs and alcohol. Number 4 holds the memories and trauma of these events, physical abuse by her stepfather, and anger/judgmental tendencies the patient internalized from her biological father. This alter stopped aging when the patient was 16 and met her husband. This alter takes control when the patient feels venerable, scared, threatened, or when the alter believes the patient is in jeopardy. For example, the patient tried to kill herself every night for the first 18 months of therapy by overdosing, but unbeknownst to the patient, #4 made the patient vomit every night leaving the patient to believe she'd failed at suicide.

In addition to #1–4 the patient has partial personality alters which are vague in appearance and manifestation and did not develop their own individual identity. Each of these came into existence during a traumatic event to hold the pain and emotion from the event and ceased to exist once the patient obtained access to the repressed memories. These partials were identified when one of the four alters would gain further insight to a past event or new memories that they had not previously possessed.

**Figure 1:**
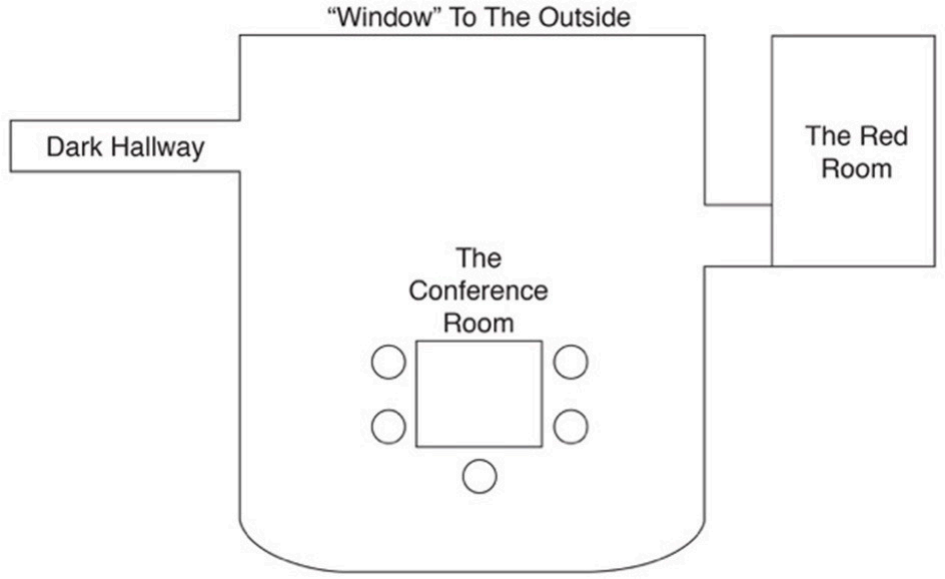
A replication of the patient's drawing of the space inside her mind. The space has a table and chairs where the four alternate personalities the host personality and would sit, interact/play with one another, and discuss who would take control and who would answer question. The Red Room is where #4 locks the other alters if they misbehave and where memories of major trauma are held. The dark hallway only appeared after the patient received ECT.

All of the alters exist in a space inside the patient's mind. Figure 1 is a schematic of what the patient drew while describing the space where the alters lived, and interacted with one another. The Red Room, which is connected to this space, has “blood flowing down its walls,” is “black inside,” and holds the most severe trauma described as streams of images of the abuse and assault that run around the top of this room. Late into therapy, it became apparent that #4 makes the patient and other alters go into The Red Room whenever she is displeased with them. The alters would converse with one another in the “conference room” about how they would answer questions and decide who would take control.

Prior to initiating medications, the patient was never able to manage her PTSD, MDD, and associated features such as passive or active suicidal ideations and psychomotor retardation. Furthermore, she exhibited cognitive deficits prior to treatment such as impaired complex attention, executive function, learning, memory, language abilities, and social cognition. As a result of these deficits and the patient's multiple identities she had trouble remembering, or no memory at all, of some therapy sessions. This hindered her improvement and was responsible for the amount of therapy session required to produce any improvement and restricted phase specific psychotherapy. She tried a myriad of medications including Lithium, Lamotrigine, Ziprasidone, Quetiapine, Lurasidone, Sertraline, Amitriptyline, Citalopram, Duloxetine, Doxepin HCl, Ketamine, Alprazolam, Clonazepam, Lorazepam, Zolpidem, and Prazosin. After 8 hospitalizations for substance abuse and suicide attempts within a 2-year period, the treating inpatient psychiatrist decided on a trial of Electroconvulsive Therapy (ECT) to reduce her suicidality.

During the most severe bout of suicidality before undergoing ECT, the patient reported the presence of psychotic features, e.g. the presence of a “demon who floated above the floor with green eyes, long fingers, and a cloak” who told her that she would be allowed to kill herself later and that she would “suffer in Hell after dying.” These hallucinations, like others she experienced in her past, was a manifestation of her constant and overwhelming guilt, her belief she deserved punishment, and her profound sense of worthlessness. The patient also had visual hallucinations of her uncle/abuser in her bedroom at night time. Furthermore, she had auditory hallucinations of the medical providers that were assisting in her treatment.

When she was hospitalized in September 2014 the patient started receiving ECT weekly until September 2016. The patient was administered Ketamine prior to the ECT procedure. The ECT parameters were unilateral d'Elia electrode placement with a pulse width 0.3 ms, frequency 120 Hz for 8 s, 800 mA current, dynamic energy 87.9 joules, and seizure duration of 34 s.

After her weekly ECT regimen, medications were consolidated to only include Doxepin HCl and Alprazolam because she was responding well to ECT. However, due to the patient's depression with psychotic symptoms, Lurasidone was added to her medication regime. Following this time frame, she was gradually moved down to ECT every other week.

While the patient was receiving ECT every other week, around November of 2016, she admitted she was starting to experience flashbacks from her childhood. At this time only the host personality existed, i.e., she couldn't see, hear, or feel the others. The patient was completely alone from the time the others disappeared in November 2016 until she began to hear #4, the strongest alter, in February of 2017 but has never seen this alter to date. However, the others haven't reappeared since November 2016. During this time, the patient said she could “feel the others missing” but hoped “they were still around, but didn't know where they were.” This was when her integration of personalities began. The patient admitted that she could truly feel and know the other's thoughts and feelings for the first time in her life. Starting in February of 2017 she experienced two to five flashbacks a day lasting from minutes to hours. With each flashback, she recalled new memories and additional details of old memories about past traumas. She also began to “feel the others pain and emotions” and began to better understand her fractured past.

To date, the patient has not seen or heard the missing personality alters and can only hear #4. She is learning details about the physical, emotional, and sexual trauma she suffered with each flash back. Currently, the patient's mood has been stabilized on Doxepin HCl, Alprazolam, and Lurasidone in addition to monthly ECT. This treatment regimen prevented suicidal ideation in the patient until her flashbacks began. To date, she occasionally commits non-suicidal self-injury and has been experiencing suicidal ideations as she learns more about the trauma from what the alters protected her.

## Background

DID is a polarizing topic within the mental health profession. According to the DSM-5, the defining feature of DID is the “disruption of identity characterized by two or more distinct personality states or an experience of possession… [with] discontinuity in sense of self and sense of agency, accompanied by related alterations in affect, behavior, consciousness, memory, perception, cognition, and/or sensory-motor functioning” (1). Other features include “recurrent gaps in recall of everyday events, important personal information, and/or traumatic events that are inconsistent with ordinary forgetting” (1). The dispute about the legitimacy of DID stems from varying theories as to the etiology and treatment of the disorder. The two predominant models are the post-traumatic model (PTM) and the socio-cognitive model (SCM) (2–5). The foundation of PTM is the belief that children dissociate/fracture to avoid the psychological consequences of severe trauma or abuse (4, 6–9), whereas SCM believes that personality alters are sociocultural constructs, independent of trauma, due to attention-seeking behavior, that is not deception, and independent of conscious effort (2, 10–12). Newer perspectives have started to integrate the two schools of thought rather than looking at PTM and SCM as mutually exclusive models (5).

Both schools of thought report the concomitant presence of depression (5, 13–15). However, the fundamental principles of PTM better explain DID associated with anxiety, MDD, and PTSD. Trauma is pervasive, especially in early development. Trauma early on in life is known to result in cognitive deficits and delays in emotional developmental (16). These deficits have their roots in the negative interactions between the child and the adult caregiver that force the child protect itself psychologically and impede the development of a healthy and fully functional personality (12, 16, 17). Developmental delays resulting from the inability of the child to form safe attachments to others and the child's lack of self-personality impede the achievement of future developmental milestones in adolescence and adulthood (16). All of these factors create an environment where dissociation is perhaps the only way for the child to cope (7, 12, 16, 18). Unfortunately, even a full personality alter is limited in how much trauma it can absorb, and if its threshold is exceeded another fracturing can occur.

With regard to the treatment of DID, those that believe in the legitimacy of the diagnosis agree that reintegration of the fractured personalities into one identity is required (3, 4, 14, 18–21). The general consensus amongst experts employees a three phase-orientated treatment approach (21). Phase 1- establishing safety, stabilization and symptom reduction, Phase 2- confronting, working through, and integrating traumatic memories, and Phase 3- integration and rehabilitation (21). The standard intervention for a DID patient is psychoanalytic therapy to facilitate personality unification with psychopharmacological agents showing minimal efficacy (6, 20, 22, 23). However, medications are frequently used to manage the comorbid disorders that tend to accompany DID. The use of ECT has been documented in the treatment of DID patients who also have MDD, but medical practitioners are weary to use it out of concern that the stress of ECT will disrupt or further damage an already fragile mind (13).

## Discussion

Herein we present a report of a patient with DID. We believe the cause of her DID was the constant psychological, physical, and sexual assault that she suffered from early childhood through adulthood. This study validates the etiological strength of PTM in explaining the origins of DID. The patient developed fully formed personality alters following severe and prolonged early developmental trauma. Subsequent full identity alters developed each time the current/most recent alter was exposed to trauma that exceeded its ability to accommodate the recurrent trauma.

It has been previously reported that individuals with DID tend to have comorbid MDD and PTSD, with a large proportion suffering from persistent suicidal ideations (12–14, 24). This case documents the intricate intertwining of DID and MDD secondary to early developmental trauma. In addition, it validates the long-term cognitive and emotional deficits previously reported in patients with early developmental trauma (5, 7, 16, 17, 24). The patient demonstrated significant cognitive deficits and psychomotor retardation. Furthermore, her severe depression and possibly the trauma within the alters likely attributed to her hallucinations.

The patient was unable to function adaptively while treated with psychotherapy and medication but with the addition of ECT she began to show significant improvement in affect and decreased suicidal ideations. It was only after the patient received a significant course of ECT that she started to experience flashbacks and reported the absence of “the others.” As the flashbacks continued the patient reported learning about and feeling what her alters felt. She even admitted to feeling bad for what the others had gone through. While there have been only a few previous reports on the efficacy of ECT for treatment of DID and other comorbid disorders (13, 25), we believe that ECT contributed in some capacity to this patient's personality integration. Despite 10 years of being in therapy taking psychotropic medications, personality integration occurred only after a course of prolonged and frequent ECT. The importance of therapy and medications during this time cannot be downplayed. These too, contributed to integration of the alters by helping the patient process losing part of herself and cope with traumatic memories. Augmentation to the patient's mood was greatly seen and concomitant remission of her psychotic symptoms with the addition of Lurasidone. Other atypical antipsychotics were not successful in managing the patient's mood because she was a diabetic and all the other medications in that drug class increased her blood sugar level. Lurasidone was the only neuroleptic that showed advantageous mood effects without increasing the patient's blood glucose levels.

We believe the efficacy of ECT was in its ability to reduce/remove the patient's psychosis, depression, psychomotor retardation, and suicidal ideation. Furthermore, ECT hindered the patients short term memory while preserving her previously unresolved trauma. As a result, this allowed the patient to process and recognize her past experiences in a safe therapeutic environment without resorting to self-destructive coping mechanisms. With the ability to process the emotional trauma, we believe the patient's trauma threshold decreased and subsequently rendered the protective properties of the alters unnecessary to allow for identity integration. Without continued ECT, therapy, and psychotropic medications there would have been a significant risk that “the others” would have come back and that the patient would not have processed the traumatic events that were held by the full personality alters to protect her.

The integration of many identities into a singular personality is an arduous process. As the memories and feelings returned to the patient, so did increasingly severe hopelessness, guilt, pain, and thoughts of self-harm. Presently, the patient is still learning to process her new memories and we hope that she will be able to persevere and be able to understand and heal from her past.

## Concluding remarks

This report alone is unable to definitively establish ECT as an effective tool for the integration of personality alters in patients with DID. However, we believe that practitioners should not avoid considering ECT for the treatment of patients with DID and comorbid severe and chronic MDD with suicidal ideation. Furthermore, we believe this case report adds to the validity of the PTM in the etiology of DID. We hope this report prompts further research into the efficacy of ECT, psychotherapy, and psychopharmacology on identity integration in DID patients.

## Ethics statement

The client of the study gave verbal and written informed consent for the publication of this case study on the assurance that all Protected Health Information would be redacted.

## Author contributions

KW is the medical student that worked with both TH and SM to compile all the information on the patient, background information, and wrote the manuscript. SM is the patient's Psychiatric Nurse Practitioner who has managed her medications and point of contact for the doctors who administered ECT. TH is the patients therapist who conducted most of the 450+ therapy sessions.

### Conflict of interest statement

The authors declare that the research was conducted in the absence of any commercial or financial relationships that could be construed as a potential conflict of interest.
